# Association of *BAX* hypermethylation with coronary heart disease is specific to individuals aged over 70

**DOI:** 10.1097/MD.0000000000014130

**Published:** 2019-01-25

**Authors:** Limei Zhang, Huihui Ji, Yi Huang, Haochang Hu, Bin Li, Yong Yang, Hang Yu, Xiaoying Chen, Wenxia Li, Fang Liu, Shi Wang, Chunming Wang, Ke Chen, Yingchun Bao, Haibo Liu, Shiwei Duan

**Affiliations:** a Department of Cardiology, Yinzhou People's Hospital, Ningbo University, Ningbo; b Medical Genetics Center, School of Medicine, Ningbo University, Ningbo; c Department of Cardiovascular Medicine, East Hospital, Tongji University School of Medicine, Shanghai, China.

**Keywords:** age, *BAX*, coronary heart disease, DNA methylation, Lp(a), smoking

## Abstract

**Introduction:**

As a member of B-cell lymphoma-2 (BCL-2) gene family, BCL-2 associated X (*BAX*) is important for cell apoptosis. In this work, we investigated the association of *BAX* promoter DNA methylation with coronary heart disease (CHD) in Han Chinese.

**Methods:**

A SYBR green-based quantitative methylation specific PCR (qMSP) was used to test *BAX* methylation levels in 959 CHD cases and 514 controls.

**Results:**

Although *BAX* methylation was not associated with CHD in the total samples, further breakdown analysis by age showed that *BAX* hypermethylation was significantly associated with CHD for individuals aged over 70 (median percentage of methylation ratio [PMR], 10.70% in cases versus (vs) 2.25% in controls, *P* =.046). Moreover, *BAX* methylation was associated with smoking and lipoprotein A (Lp(a)) for individuals aged over 70 (CHD: smoking *P* = .012, Lp(a) *P* = .001; non-CHD: smoking *P* = .051, Lp(a) *P* = .004). Further analysis of Gene Expression Omnibus (GEO) and The Cancer Genome Atlas (TCGA) data showed *BAX* expression was upregulated by 5-aza-2’-deoxycytidine demethylation agent (fold = 1.66, *P* = .038) and inversely correlated with *BAX* methylation (r = −0.428, *P* = 8E-05).

**Conclusions:**

Our study supported that *BAX* hypermethylation might contribute to CHD risk via downregulation of *BAX* expression for individuals aged over 70.

## Abstract 2

Supplemental Digital Content is available in the text

## 1 Introduction

Nearly 7 million annual deaths are attributable to coronary heart disease (CHD).^[1]^ The CHD is characterized by the stenosis or occlusion of the coronary artery.^[2]^ The CHD is a complex disease accompanied by several complications such as elevated cholesterol levels and cigarette smoking.^[3]^

The DNA methylation is a crucial epigenetic marker regulating gene expression.^[4]^ It often occurs at a CpG site where a cytosine is directly followed by a guanine in the DNA sequence. Hypermethylation of gene promoter could induce transcriptional silencing^[5,6]^ and often involve in the development of diseases.^[7]^

The B-cell lymphoma-2 (BCL-2) family proteins are famous for the capacity for the regulation of programmed cell death.^[8]^ The BCL-2 can prevent cell apoptosis and its over-expression may promote cancer cell survival.^[9]^ Human BCL-2 associated X (*BAX)* is the 1st death-promoting member in BCL-2 family.^[10]^ The *BAX* can suppress cell apoptosis process.^[8]^ The expression level of *BAX* was important for heart disease including CHD. Overexpressed *BAX* was found to accelerate myocyte apoptosis during ischemia and reperfusion.^[11,12]^

Although no association of *BAX* methylation and CHD was discovered between 205 CHD patients and matched controls in an early study,^[13]^ we carried out the present study among 959 CHD cases and 514 controls to assess the relationship between *BAX* methylation and CHD.

## 2 Materials and methods

### 2.1 Ethics statement

Institutional review ethics board approval was obtained from Yinzhou Peoples Hospital and Ningbo No.1 Hospital. All the individuals were formally informed, and the written informed consents were obtained from all the participants or their guardians.

### 2.2 Patient selection

The present study enrolled Chinese patients with CHD from Yinzhou Peoples Hospital and Ningbo No.1 Hospital. The patients were diagnosed with the angiographic evidence that coronary artery stenosis was greater than 50% or a history of prior angioplasty or coronary artery bypass surgery. The inclusion criteria were described previously.^[14,15]^ The peripheral blood samples were drawn from 959 CHD (635 males and 324 females, median age: 62 years) patients and 514 healthy controls (291 males and 223 females, median age: 60 years) for DNA methylation assay. About 50% and 18% of participators were accompanied by hypertension and diabetes, respectively. Among them, about 33% of participators were smokers. The clinical indexes including low density lipoprotein (LDL), total cholesterol (TC), high density lipoprotein (HDL), apolipoprotein A1 (ApoA1), apolipoprotein B (ApoB), apolipoprotein E (ApoE) and lipoprotein A (Lp(a)) were measured using standard protocols at the time of collecting DNA samples. Blood samples were stored at −80°C with EDTA anticoagulant tube.

### 2.3 DNA extraction, bisulfite conversion and quantitative methylation specific PCR (qMSP)

The procedures of DNA extraction from peripheral blood and the subsequent bisulfite conversion were the same as previously described.^[16]^ The *BAX* methylation was measured by qMSP, and the percentage of methylation ratios (PMRs) was applied to represent gene methylation levels.^[17,18]^ The details of qMSP were available in our previous publications.^[19–22]^ The forward and reverse primer sequences of *BAX* were 5’-GAAGGTATTAGAGTTGCGATT-3’ and 5’-CCAATAAACATCTCCCGATAA-3’, respectively. The forward and reverse primer sequences of *ACTB* were 5’-TGGTGATGGAGGAGGTTTAGTAAGT-3’ and 5’-AACCAATAAAACCTACTCCTCCCTTAA-3’, respectively.

### 2.4 Data-mining of the online datasets

The Gene Expression Omnibus (GEO) data sets from the National Center for Biotechnology Information (NCBI) was used to browse for gene expression microarrays data. The cells were collected before and after 5’-aza-2’-deoxycytidine (5’-AZA) treatment, respectively. The *BAX* expression values in cells were acquired from an Illumina HumanRef-8 v3.0 expression beadchip. The detailed data was under accession No.GSE38823 in GEO data sets.^[23]^ The *BAX* methylation data and its mRNA expression values were retrieved from (The Cancer Genome Atlas) TCGA database.

### 2.5 Statistical analysis

A *P* value < .05 was considered to be statistically significant. Categorical data were shown as number and percentages, and then analyzed using Pearson Chi-squared or Fisher's exact test (when expected value of any cell is less than 5). Nonparametric test was used to compare the differences of *BAX* methylation between CHD cases and normal controls. The correlations between clinical indexes and *BAX* methylation were examined by the Spearman correlation.

## 3 Results

**Figure 1:**
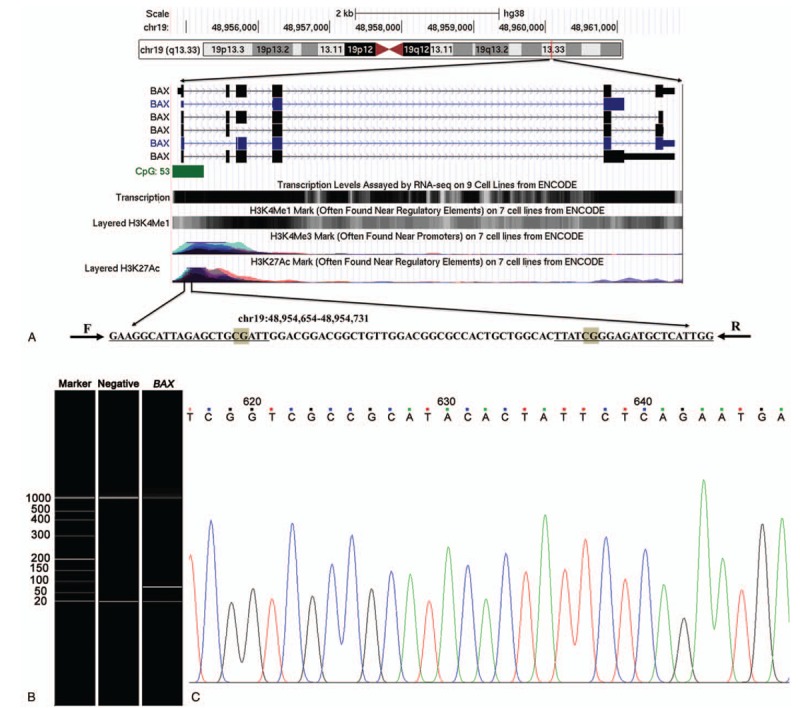
The target sequence in *BAX* methylation assay. The 2 tested CpG dinucleotides in *BAX* promoter. F and R were forward primer and reverse primer, respectively. Verification of the qMSP product length by capillary electrophoresis; Sequencing validation. BAX = BCL-2 associated X, qMSP = quantitative methylation specific PCR.

A fragment from CpG island of *BAX* promoter was measured in this study (Fig. 1). However, there was no significant difference of *BAX* methylation between CHD cases and controls (*P* = .384, Table 1). As shown in Table 1, a significant association was found between age and CHD [OR (95% CI) = 1.040 (1.025–1.055), *P* = 7E-8]. Moreover, our results suggested that smoking and diabetes could increase the risk of CHD [smoking, *P* = .026, OR (95% CI) = 1.453 (1.046–2.020); diabetes, *P* = .002, OR (95% CI) = 1.822 (1.256–2.643)]. Besides, there were significant associations between CHD and the levels of biochemical indexes including LDL, triglyceride and Lp(a) [LDL, OR (95% CI) = 1.727 (1.171–2.547), *P* = .006; triglyceride, OR (95% CI) = 1.291 (1.084–1.538), *P* = .004; Lp(a), OR (95% CI) = 0.998 (0.997–0.999), *P* = .001]. The associations between *BAX* methylation and clinical indexes were also analyzed (Supplemental Table 1).

**Table 1:**
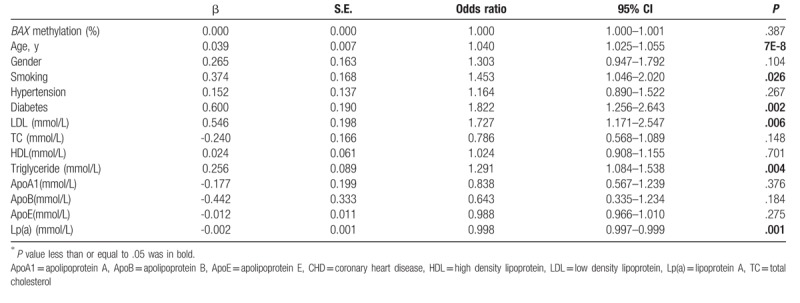
Multiple logistic regression analyses between phenotypes and coronary heart disease (CHD)^∗^.

Since age was one of the most influential factors on alteration of DNA methylation,^[24]^ we further performed a breakdown analysis by age. As shown in Table 2, *BAX* hypermethylation was significantly associated with CHD among individuals aged over 70 (median PMR, 10.70% versus 2.25%, *P* = .046). For individuals aged over 70, *BAX* hypermethylation was found to be associated with smoking which was a risk factor of CHD (CHD: *P* = .012; non-CHD: *P* = .051, Table 3). Moreover, an inverse association was observed between *BAX* methylation and triglyceride in CHD cases aged over 70 (r = −0.153, *P* = .040, Table 4). And among individuals aged over 70, *BAX* methylation was inversely related to Lp(a) (cases, r = −0.266, *P* = .001; r = −0.411, *P* = .004, Table 4). Therefore, our results indicated that the association of *BAX* hypermethylation with CHD was specific to individuals aged over 70.

**Table 2:**
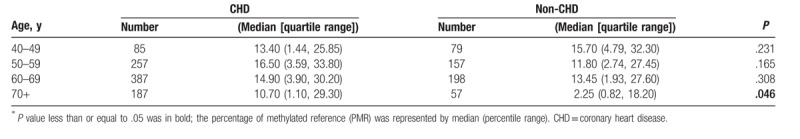
The comparisons of *BAX* methylation between coronary heart disease (CHD) and non-CHD by age^∗^.

**Table 3:**
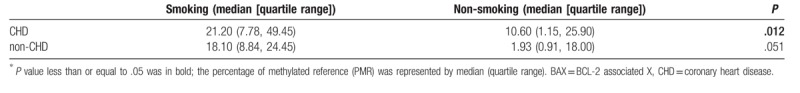
The comparisons of BCL-2 associated X (*BAX*) methylation between smoking and non-smoking among individuals aged over 70^∗^.

**Table 4:**
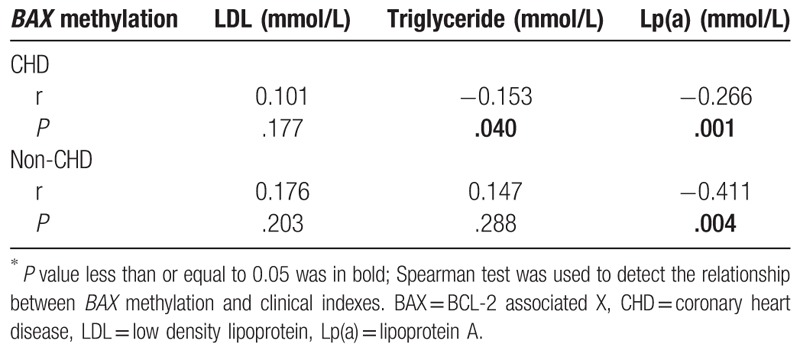
Associations of BCL-2 associated X (*BAX*) methylation and biochemical indexes among individuals aged over 70^∗^.

As shown in Table 5, smoking was significantly associated with BAX methylation in individuals aged over 70 and all subjects (over 70, r = 0.228, *P* = .001, total, r = 0. 056, *P* = .045). Triglyceride was related to BAX methylation in aged 50–59 (r = −0.115, *P* = .022). Besides, there were significant associations between Lp(a) and BAX methylation in aged 50–59, 60–69, ≥ 70, and all subjects, respectively (50–59, r = −0.261, *P* = 6.E-07, 60–69, r = −0.253, *P* = 2.E-08, ≥ 70, r = −0.384, *P* = 2.E-08, total, r = −0.274, *P* = 2.E-22).

**Table 5:**
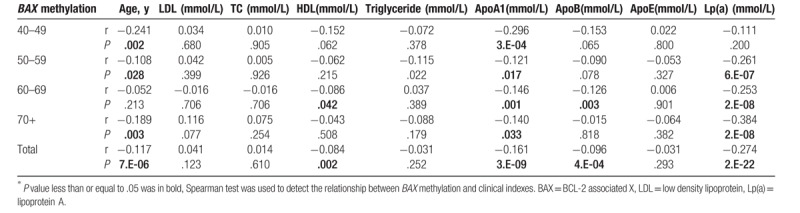
Associations of BAX = BCL-2 associated X (*BAX*) methylation and biochemical indexes among individuals^∗^.

**Figure 2:**
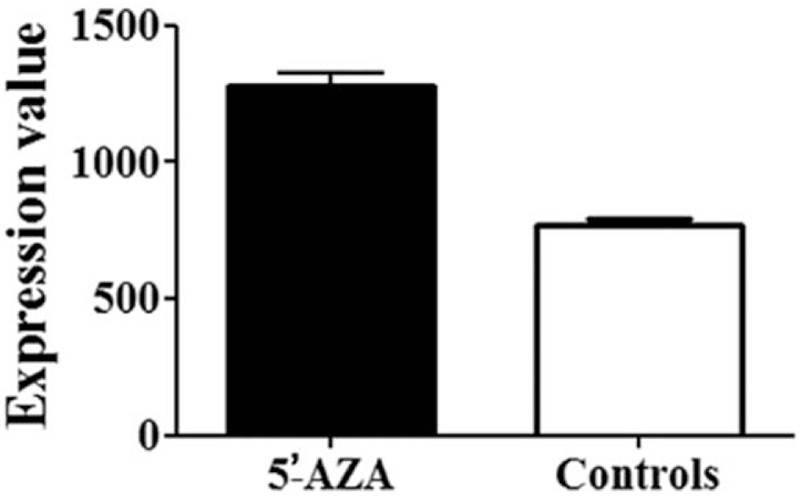
Demethyaltion agent significantly increased *BAX* expression. 5’-AZA denoted the cells treated by 5’-AZA. Controls denoted the cells without the treatment. Y-axis was the level of *BAX* expression. The detailed data was retrieved from accession No.GSE38823 in GEO database. BAX = BCL-2 associated X, 5’-AZA = 5’-aza-2’-deoxycytidine, GEO = Gene Expression Omnibus.

**Figure 3:**
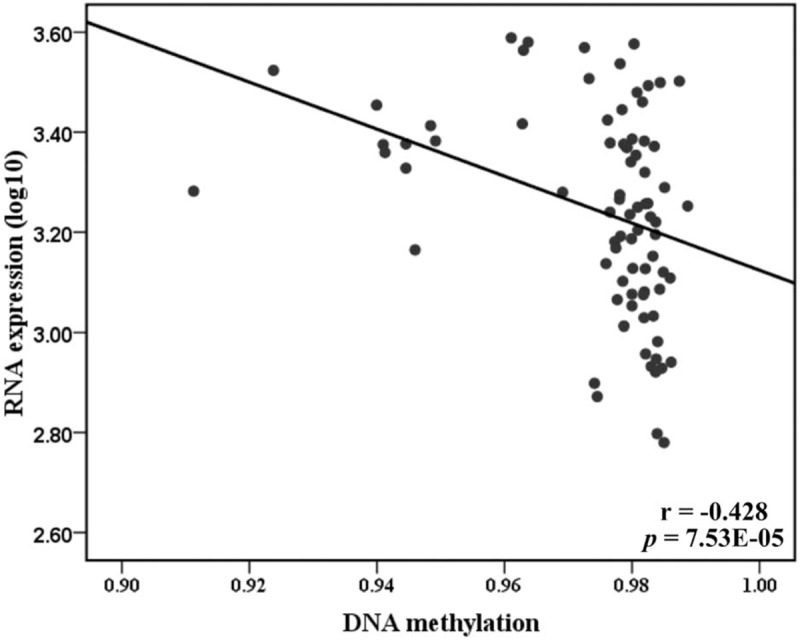
The *BAX* methylation inversely correlated with *BAX* expression. Pearson correlation were performed for the correlation test. The *BAX* methylation and its mRNA expression values were retrieved from TCGA database. BAX = BCL-2 associated X, TCGA = The Cancer Genome Atlas.

The GEO data analysis showed a significantly higher level of *BAX* in cells after 5’-AZA demethylation agent treatment (fold = 1.66, *P* = .038, Fig. 2). The TCGA data analysis indicated *BAX* methylation was inversely associated with *BAX* expression (r = −0.428, *P* = 8E-5, Fig. 3). Therefore, we hypothesized that *BAX* hypermethylation might contribute to CHD among individuals aged over 70 via its downregulation of *BAX* expression.

## 4 Discussion

Our results demonstrated the association of *BAX* hypermethylation with CHD was specific to individuals aged over 70. Among individuals aged over 70, *BAX* hypermethylation was associated with smoking and lower plasma Lp(a) level. The *BAX* expression was induced by methylation inhibitor and further online data mining found that *BAX* expression was inversely related to its methylation.

The *BAX* was considered to be the most important apoptosis-inhibition gene.^[25–27]^ Abnormal *BAX* expression was shown to be associated with heart disease.^[28,29]^ Our study suggested that *BAX* methylation was significantly higher in CHD patients over 70. Moreover, further analyses of GEO and TCGA data showed that *BAX* hypermethylation would suppress its RNA expression. Our study showed that the *BAX* hypermethylation might contribute to CHD among individuals aged over 70 via its downregulation of *BAX* expression. Further studies were needed to discover the role of the *BAX* methylation in CHD.

The *BAX* is an important player in apoptosis and encodes a variety of transcripts. The *BAX* is associated with developmental, cancer and age-related changes in apoptosis.^[27]^ Inactivation of *BAX* in mice prolongs fertility potential and minimizes age-related health problems.^[30]^ It is unclear how these processes are related to human aging, but the role of *BAX* in human aging is possible. In the present study, *BAX* methylation was significantly decreased in Non-CHD individuals aged over 70 while the CHD group in the same age seems to keep the methylation level. Previous studies have shown that statins can promote epigenetic-based control in CVD prevention through histone modifications.^[31]^ In addition, atorvastatin inhibits neointimal formation by inducing p16 expression by inducing DNA methylation in the p16 promoter region.^[32]^ Thus, the decreased *BAX* methylation in the CHD group may be rescued by drug therapy or other treatment-related factors. Further research was needed to confirm this hypothesis.

Ageing is a risk factor for multiple chronic diseases.^[33]^ Ageing impacts all organ systems leading to decreased functionality and eventual death.^[34]^ Accumulating evidence has shown that DNA methylation changes are associated with ageing and its related phenotypes.^[24,35–38]^ Our study found that *BAX* methylation was associated with CHD only among individuals aged over 70, adding a new ageing-related clue of CHD.

Tobacco smoking was associated with significant modifications of gene methylation.^[39–41]^ Tobacco smoking was shown to increase the risk of cardiovascular disease.^[42,43]^ A mice experiment showed *BAX* expression could be induced by exposure to smoking in oocytes.^[8]^ Furthermore, ovarian damage caused by smoking could prevented by *BAX* inactivation.^[44]^ Our study found that *BAX* methylation was associated with smoking among individuals aged over 70. The *BAX* methylation might be involved in the regulation of cell apoptosis caused by smoking.

In the recent years, the relationship between lipid metabolism and CHD have been discovered by previous studies.^[45,46]^ The ratio of TG/HDL-C was proportional to the severity degree of CHD.^[47]^ The *BAX* methylation level would deceased by oxidized LDL and then lead to cell apoptosis. Inconsistent with a previous study,^[48]^ our results showed *BAX* methylation was inversely related to plasma Lp(a), suggesting that *BAX* hypermehtylation correlated with a lower level of plasma Lp(a), a protective factor of CHD shown in the present study. Further studies covering large samples should be done to explore the detailed relationship between CHD and Lp(a).

A recent study showed that the levels of TC, LDL, non-HDL and ApoB were significantly higher in patients with myocardial infarction than in the control group, and these lipid levels showed a downward trend with age (27737874). Meanwhile, the levels of ApoA1 and HDL in patients with myocardial infarction were significantly lower than those in the control group, and the levels of both increased significantly with age (27737874). In high-fat-fed mice, lipid accumulation in the hippocampal CA3 region was observed, and plasma lipid levels (including triglycerides, TC, LDL, and HDL) were significantly increased, accompanied by an increase in *BAX* expression (28000893). Our study found that *BAX* methylation levels were inversely correlated with age, ApoA1, and Lp(a) in different age groups. Taken together, we hypothesized that changes in *BAX* methylation levels were likely to have an effect on the pathogenesis of CHD under the combined effects of age and lipid levels.

There are some limitations in the present study. Firstly, since our findings from a case-control study are just correlative and possibly not causal, the case-effect relationship between *BAX* methylation and CHD remains unclear. Therefore, a more convincing method such as cohort study is needed in the future. Secondly, only 2 CpG sites from CpG island has been selected to represent the whole gene of *BAX*. Thirdly, we only have measured *BAX* methylation in peripheral blood samples. However, the DNA methylation profile may vary among different tissues more or less. Further studies are needed to confirm our findings in other tissues.

In summary, our research found that *BAX* hypermethylation might contribute to CHD among individuals aged over 70 years.

## Author contributions

The experiment was designed by HL and SD. The patients’ information was collected by LZ, SW, CW, YB, YH and KC. The experiment was performed by BL, YY, HY and XC. The data was analyzed by WL and FL. The draft was written by HJ and HH.

**Conceptualization:** Haibo Liu, Shiwei Duan.

**Data curation:** Limei Zhang, Yi Huang, Bin Li, Yong Yang, Yu Huang, Wenxia Li, Chunming Wang, Ke Chen.

**Formal analysis:** Yong Yang, Yu Huang, Xiaoying Chen, Fang Liu, Yingchun Bao.

**Investigation:** Bin Li, Xiaoying Chen, Wenxia Li, Haibo Liu.

**Methodology:** Haochang Hu, Yong Yang, Xiaoying Chen, Shi Wang, Chunming Wang, Yingchun Bao.

**Project administration:** Yingchun Bao.

**Resources:** Shi Wang, Chunming Wang.

**Software:** Fang Liu.

**Writing – original draft:** Huihui Ji, Haochang Hu.

**Writing – review & editing:** Shiwei Duan.

Shiwei Duan orcid: 0000-0001-7682-2877.

## Supplementary Material

Supplemental Digital Content
