# Supplementary material for: Association of BAX hypermethylation with coronary heart disease is specific to individuals aged over 70
Source: Medicine (Baltimore). 2019 Jan 25;98(4):e14130. doi: 10.1097/MD.0000000000014130 (PMC6358363; doi:10.1097/MD.0000000000014130)
Supplement: Supplemental Digital Content [file medi-98-e14130-s001.doc]

Supplemental Table 1. The associations between BAX methylation and clinical indexes*.

|  |  | age (years) | LDL (mmol/L) | TC (mmol/L) | HDL(mmol/L) | Triglyceride (mmol/L) | ApoA1(mmol/L) | ApoB(mmol/L) | ApoE(mmol/L) | Lp(a) (mmol/L) |
| --- | --- | --- | --- | --- | --- | --- | --- | --- | --- | --- |
| BAX methylation | r | -0.117 | 0.041 | 0.014 | -0.084 | -0.031 | -0.161 | -0.096 | -0.031 | -0.274 |
|  | p | **7E-06** | 0.123 | 0.61 | **0.002** | 0.252 | **2E-09** | **4E-04** | 0.293 | **2E-22** |

**p* value less than or equal to 0.05 was in bold; Spearman test was used to detect the relationship between BAX methylation and clinical indexes; LDL: Low density lipoprotein; TC: Total cholesterol; HDL: High density lipoprotein; ApoA1: Apolipoprotein A; ApoB: Apolipoprotein B; ApoE: Apolipoprotein E; Lp(a): Lipoprotein A.
